# Hidradenitis suppurativa electronic medical record (EPIC) tool: A convenient Tool for hidradenitis suppurativa specialty clinics

**DOI:** 10.1016/j.jdin.2024.09.020

**Published:** 2025-01-21

**Authors:** Zachary Wendland, Katelyn J. Rypka, Lindsey Greenlund, Claire Herzog, Bisma Khalid, Jennifer Steinhaus, Noah Goldfarb

**Affiliations:** a Department of Dermatology, Minneapolis Veterans Affairs Health Care System, Minneapolis, Minnesota; b Department of Dermatology, University of Minnesota, Minneapolis, Minnesota; c School of Medicine, University of Minnesota, Minneapolis, Minnesota; d Fairview Health Services, M Health Fairview, Minneapolis, Minnesota; e Department of Medicine, Minneapolis Veterans Affairs Health Care System, Minneapolis, Minnesota; f Department of Medicine, University of Minnesota, Minneapolis, Minnesota

**Keywords:** assessment, electronic, EPIC, hidradenitis, improvement, measures, medical, outcome, qualitative, quality, records, severity, suppurativa, tool

*To the Editor:* We express our appreciation for the insightful article by Shih et al,^1^ which highlights the importance of establishing hidradenitis suppurativa specialty clinics (HSSCs) as valuable resources for patients with this complex condition. We would like to contribute to the discussion by suggesting the inclusion of a specific tool available within the EPIC electronic medical record (EMR) system that can enhance the care provided in HSSCs.

As the article points out, there are various logistical considerations involved in establishing an HSSC, including clinic scheduling, staff training, and coordination of care. In addition to the recommendations provided, we believe that incorporating the use of the HS Assessment Tool within the EPIC EMR system can greatly benefit dermatologists and healthcare professionals caring for HS patients. Most hidradenitis suppurativa (HS) severity instruments and outcome measures require time-consuming calculations.^2^^,^^3^ Computing and trending these scores by hand is incongruent with the most fast-paced ambulatory clinical settings. A survey of HS clinicians found that providers preferred severity assessments that were “simple, easy, clear” and “fast, convenient, practical.”^3^

The initial build of our “HS Assessments” tool was created in 2019 and went live within Fairview Health Services in 2020. Since its launch, we have implemented over 2 years’ worth of edits and revisions from our team and from real-time users. The EPIC HS tool includes 3 flowsheets: HS Nurse Assessment (Fig 1), HS Provider Assessment (Fig 2), and HS Bandage.

**Fig 1:**
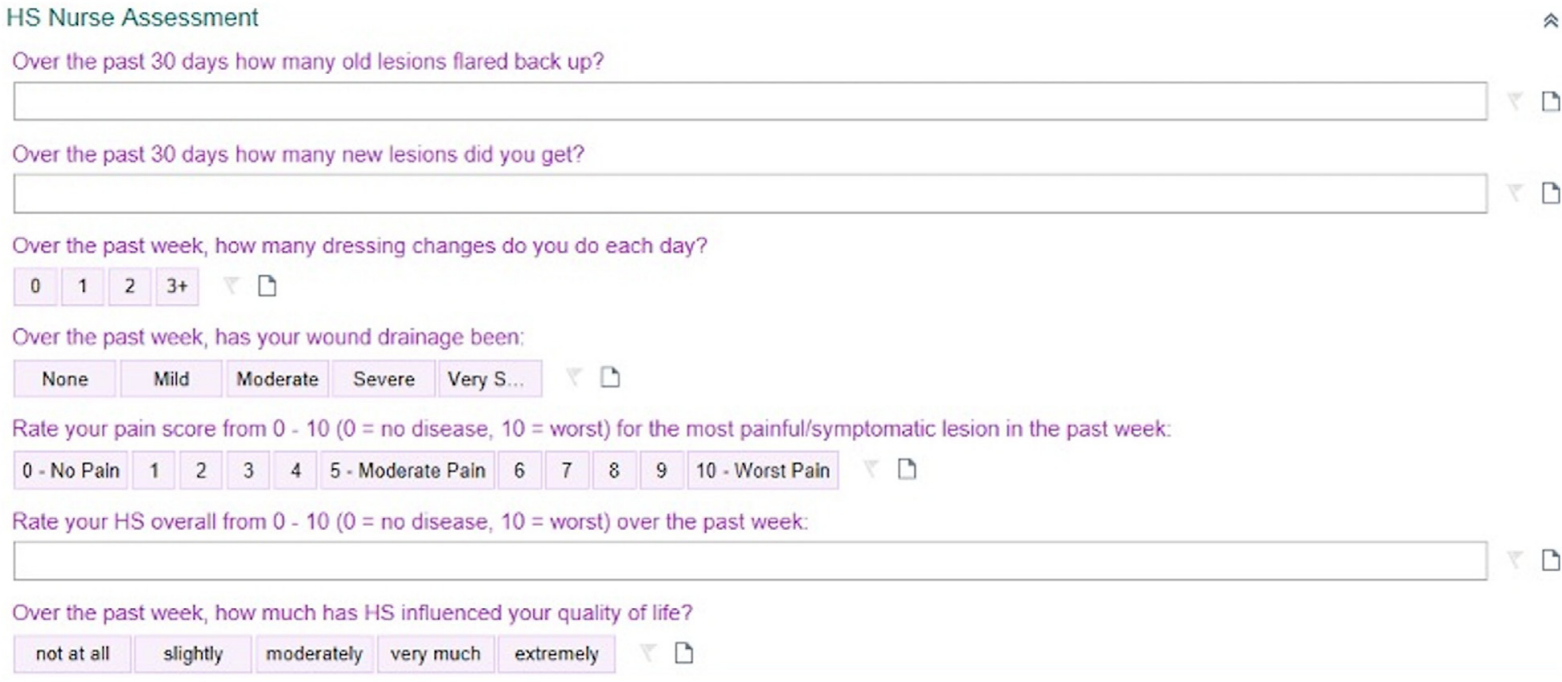
HS nursing assessment. *HS*, Hidradenitis suppurativa.

**Fig 2:**
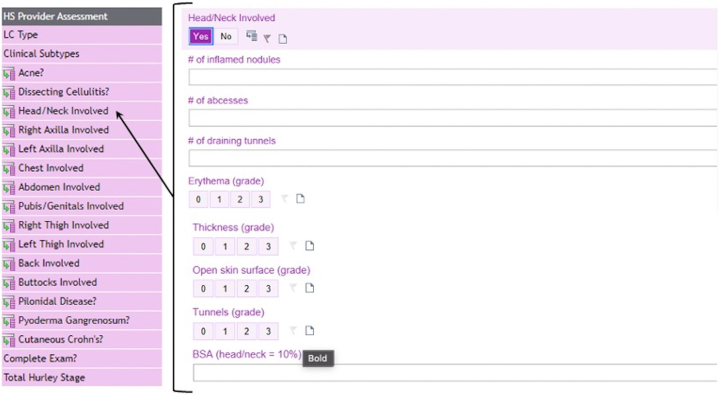
HS provider assessment. *HS*, Hidradenitis suppurativa; *BSA*, body surface area.

The HS Assessment Tool is designed to streamline the management of HS patients by providing a comprehensive and user-friendly interface within the EMR system. It offers several features that optimize clinic flow and improve patient outcomes.

The nurse assessment covers drainage, pain, and the validated patient global assessment. In the provider assessment, users enter clinical subtypes, cutaneous comorbidities, lesion counts, Hurley stage, and Hidradenitis Suppurativa Severity and Activity Index-Revised variables. The tool calculates total counts (inflammatory nodules, abscesses, draining tunnels, abscess-nodules), total Hidradenitis Suppurativa Severity and Activity Index-RevisedR, and the International Hidradenitis Suppurativa Severity Score (IHS4). The HS Bandage assessment simplifies wound management by expediting the ordering of supplies by inputing orders in a succinct table outlining wound type, wound description, bandage type, bandage size, and frequency of bandage changes.

This tool captures several validated patient and physician-reported measures and complements outpatient clinic flow. Over the past year, from July 2020 to July 2022 it was used 1085 times with over 12,000 entries. Providers may request access from their EPIC information technology department under the name “HS Assessments” associated with M Health Fairview.

By incorporating the tool, HSSCs can further optimize their clinical workflows. The integration of an electronic tool specifically designed for HS management promotes efficiency, and consistency.

In conclusion, we commend Toledo et al^1^ for their valuable contribution to the field of HS care. We suggest considering the addition of the tool within the EPIC EMR system to complement the recommendations outlined in the article. By leveraging technology to its fullest potential, we can further advance the care provided to our patients with HS.

## Conflict of interest

Dr Goldfarb has participated in clinical trials with Abbvie, Pfizer, Chemocentrix, and DeepX Health, and served on advisory boards and consulted for Novartis and Boehringer Ingelheim. Drs Wendland, Rypka, Greenlund, Herzog, Khalid, and Author Steinhaus have no conflicts of interest to declare.
